# Impact of blueberry extract on hematological response in phenylhydrazine-induced hemolytic anemia

**DOI:** 10.1016/j.htct.2025.103744

**Published:** 2025-02-26

**Authors:** Daniela Drosdowski, Patrick Türck, Silvio Tasca, Gabriel de Lima Rosa, Edson Fernando Muller Guzzo, Sara Elis Bianchi, Adriana Simon Coitinho, Cristina Campos Carraro, Adriane Belló-Klein, Alexandre Luz de Castro, Valquiria Linck Bassani, Alex Sander da Rosa Araujo

**Affiliations:** Universidade Federal do Rio Grande do Sul (UFRGS), Porto Alegre, RS, Brazil

**Keywords:** ROS, Blueberry, Inflammation, IL-10, Hemolysis

## Abstract

The objective of this study was to explore the therapeutic effect of blueberries on hematological parameters, oxidative stress, and interleukin-10 levels in acute hemolytic anemia induced by the administration of an intraperitoneal injection of 40 mg/kg phenylhydrazine. Male Wistar rats were divided into three groups: Control, anemia (PHZ), and anemia plus blueberries (PHZ+BB). Blueberries were administered via oral gavage (250 mg/day). The erythrocyte osmotic fragility, splenomegaly, iron metabolism, hematological analysis, reactive oxygens species, sulfhydryl group, and interleukin-10 levels were evaluated. The erythrocyte osmotic fragility (in 0.85% and 0.55% sodium chloride solution) and spleen weight-to-body weight ratio (∼400%) were elevated in the PHZ and PHZ+BB Groups compared to the controls (*p*-value < 0.05). Increased transferrin and reactive oxygens species levels were found in the PHZ (15%) compared to the Control Group (*p*-value < 0.05). There was an immune inflammatory response in the PHZ Group due to increases in the total leukocyte (300%), lymphocyte (100%), and neutrophil (400%) counts compared to the Control Group (*p*-value < 0.05); the PHZ Group showed increased interleukin-10 levels (100%) compared to the Control Group (*p*-value < 0.05). Blueberries showed a partial protective effect on these parameters, since there were lower neutrophil and lymphocyte counts and diminished interleukin-10 levels in the PHZ+BB Group compared to the PHZ Group (*p*-value < 0.05). In addition, blueberries increased sulfhydryl group levels (*p*-value < 0.05). These data suggest a protective role of blueberries against inflammatory response and oxidative stress in an acute hemolytic anemia model.

## Introduction

Anemia is a global health issue that affects a significant portion of the population, particularly preschool-age children.^1^ It is characterized by low hemoglobin levels and can lead to various symptoms and complications.^2^ Hemolytic anemia is a specific type of anemia characterized by the destruction of red blood cells. It can be caused by various factors including toxins, infections, and autoimmune reactions.^3^

Phenylhydrazine (PHZ) is commonly used to induce hemolytic anemia in animal models owing to its ability to cause oxidative damage to red blood cells. In this context, reactive oxygen species (ROS) are harmful molecules that can cause damage to the developing red blood cells.^4^ Low levels of sulfhydryl groups, a key antioxidant, and elevated levels of ROS support the involvement of oxidative stress in the development of hemolysis and ineffective erythropoiesis.^5^ Disruption of redox homeostasis can trigger apoptotic signaling pathways and induce erythrocyte death.^6^^,^^7^

In this context, blueberries (*Vaccinium myrtillus* L.) are known for their health-protective attributes, such as anti-inflammatory, antioxidant, and anticancer properties.^8^, ^9^, ^10^ A high phenolic compound content, which is a secondary plant metabolite, has beneficial effects in preventing diseases attributed to the production of free radicals. Turck et al. showed the anti-apoptotic effect of blueberry extract on H9c2 cardiac cells exposed to a norepinephrine-induced oxidative stress model, indicating the cardioprotective action of this compound.^11^ In view of that, phenolic compounds from blueberries can react with the cell membranes and protect them against oxidative stress, avoiding hemolysis-induced cell death.^12^

Nevertheless, studies that explore the therapeutic impact of using blueberry extract in anemic conditions are scarce. The aim of this study was to investigate the potential therapeutic effect of blueberries on phenylhydrazine-induced hemolytic anemia in rats, focusing on the impact on hematological parameters and inflammatory markers.

## Methods

Male Wistar rats with an average age of 21 days, from the Center for Reproduction and Experimentation of Laboratory Animals (CREAL), were used. Animals were kept in the Animal House of the Pharmacology Department of the Federal University of Rio Grande do Sul (UFRGS) at room temperature (22 °C ± 2 °C) with a 12-hour light-dark cycle and free access to water and food. All procedures were approved by the Animal Care and Use Committee at the UFRGS (protocol number: 45500). All experimental groups were excluded from hematological analysis (two animals).

### Experiment design

According to the randomization method, the animals were numbered from 1 to 24 and divided into three groups: control animals received saline via gavage and intraperitoneally; anemia (PHZ Group) - received phenylhydrazine (intraperitoneal 40 mg/kg for three consecutive days) and saline via gavage; anemia plus blueberries (PHZ+BB Group) – received blueberry extract via gavage (250 mg/day for two weeks prior to anemia induction) and phenylhydrazine (intraperitoneal 40 mg/kg for three consecutive days). At the end of the experimental protocol, the animals were euthanized with an intraperitoneal anesthetic overload of ketamine (270 mg/kg), followed by guillotine decapitation. Both spleen and blood were collected for morphometric and laboratory analyses. Blueberry extract was analyzed as described by Turck et al.^13^

### Erythrocyte osmotic fragility

The evaluation was performed according to the adapted method of Parpart^14^ and Jain,^15^ which measures the stability of erythrocytes in sodium chloride solutions at different concentrations (0.85% and 0.55%). Minimum erythrocyte resistance, maximum erythrocyte resistance, and average corpuscular fragility were determined.

### Morphometric assessment

Body weight (BW) and spleen weight (SW) were measured to determine the level of splenomegaly based on the SW/BW ratio.

### Assessment of iron metabolism

Serum iron, unsaturated iron binding capacity (UIBC), transferrin, and total iron-binding capacity (TIBC) were evaluated using an Abbott kit.

### Hematological analyses

Erythrocyte, platelet, and leukocyte counts were automatically determined (Procyte Dx, IDEXX Lab). Optical microscopy analysis by automated measurement was used to evaluate the cells and determine the leukocyte differential. These analyses were performed in collaboration with the Veterinary Clinical Analysis Laboratory of UFRGS.

### Cell injury markers

Tissue injury markers, such as lactate dehydrogenase (LDH - Abbott), interleukin-10 (IL-10), and bilirubin (Abbott), were evaluated using kits suitable for these analyses.

### Oxidative stress evaluation

Plasma was subjected to a reaction with 2′,7′-dichlorofluorescein (DCF) to determine ROS. Results are expressed in pmol of DCF/mg of protein.^16^ The total thiol content was measured by the reaction with Ellman's Reagent (DTNB). The total sulfhydryl concentration was expressed as nmol TNB/mg protein.^17^

### Statical analysis

Data were subjected to a normality test (Shapiro-Wilk Test). Parametric data were analyzed using one-way ANOVA complemented by the Tukey test to compare groups. The Kruskal-Wallis test complemented by Dunn's post-hoc test was used to compare non-parametric data. The results are expressed as means ± standard deviation or medians ± interquartile range. GraphPad Prism 8.0.2 for Windows was used for data analyses. Data were considered statistically significant at *p*-values < 0.05. The GraphPad Prism program's Grubbs test was used to determine outlier results, which were removed from statistical analyses.

## Results

### Phenylhydrazine induces increased susceptibility to hemolysis in isotonic and hypotonic media, and splenomegaly

**Figure 1:**
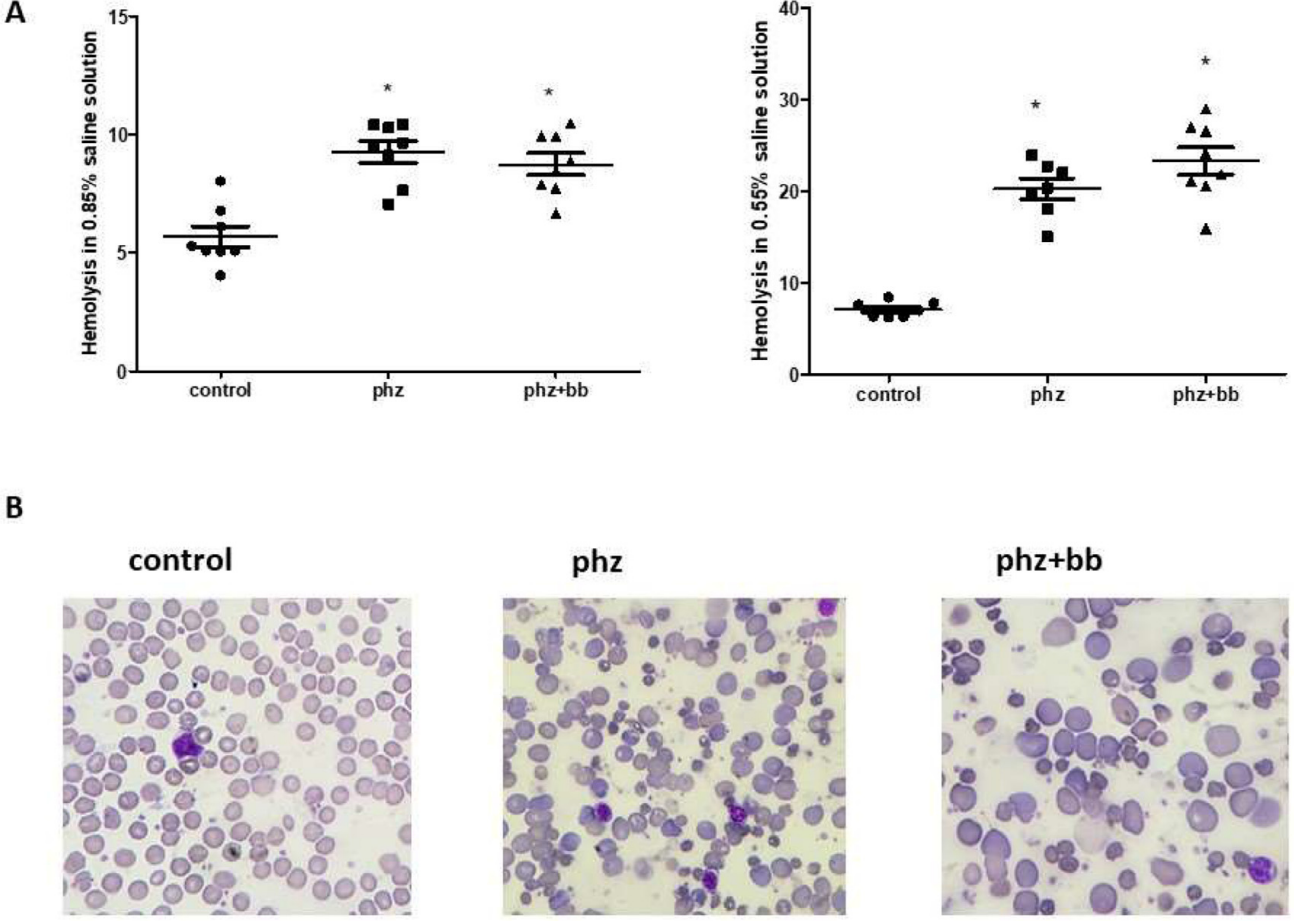
(A) Hemolysis in isotonic and hypotonic media (B) blood morphologic evaluation. Kruskal-Wallis test complemented with Dunn's post-hoc test; results expressed as medians ± interquartile range. Experimental groups: Control, anemia (PHZ), and anemia plus blueberries (PHZ+BB Group). (*) Significant difference compared to the Control Group.

**Figure 2:**
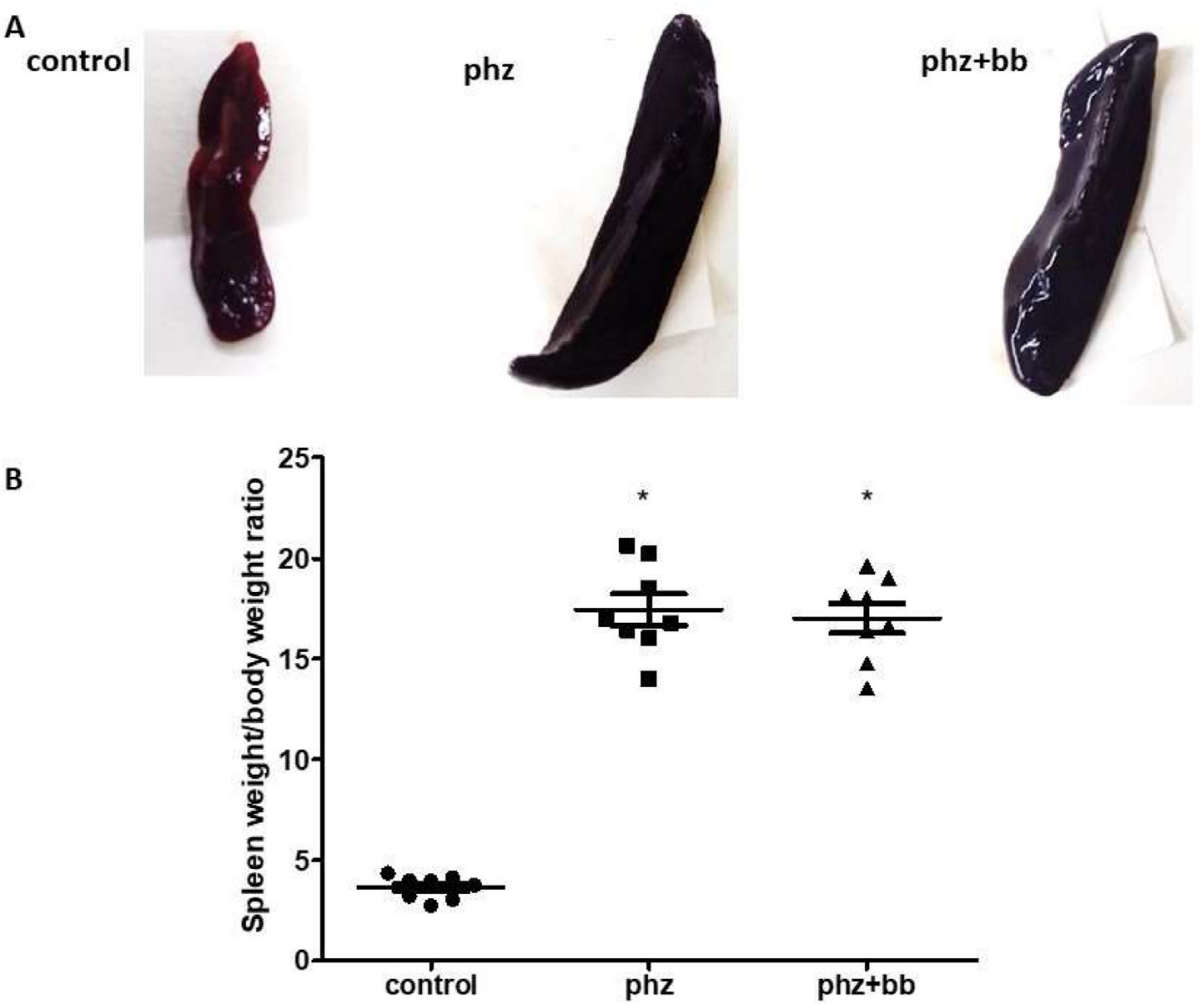
(A) Representative image of spleen (B) Splenomegaly index. Experimental groups: Control, anemia (PHZ), and anemia plus blueberries (PHZ+BB Group). Kruskal-Wallis test complemented with Dunn's post-hoc test; results expressed as medians ± interquartile range. (*) Significant difference compared to the Control Group.

The PHZ and PHZ+BB Groups showed a higher level of hemolysis in 0.85% and 0.55% sodium chloride solutions compared to the Control Group (*p*-value < 0.05). According to the blood morphologic evaluation, the presence of polychromatophilia, macrocytosis, anisocytosis, erythrocyte ghosts, and macroplatelets was higher in the PHZ and PHZ+BB Groups than in the Control Group (Figure 1A and 1B). Splenomegaly was observed in the PHZ and PHZ+BB Groups: the spleen was approximately four times larger in these groups than in the Control Group (Figure 2A and 2B).

### Phenylhydrazine-induced hemolytic crises elevate transferrin and total iron-binding capacity levels

**Figure 3:**
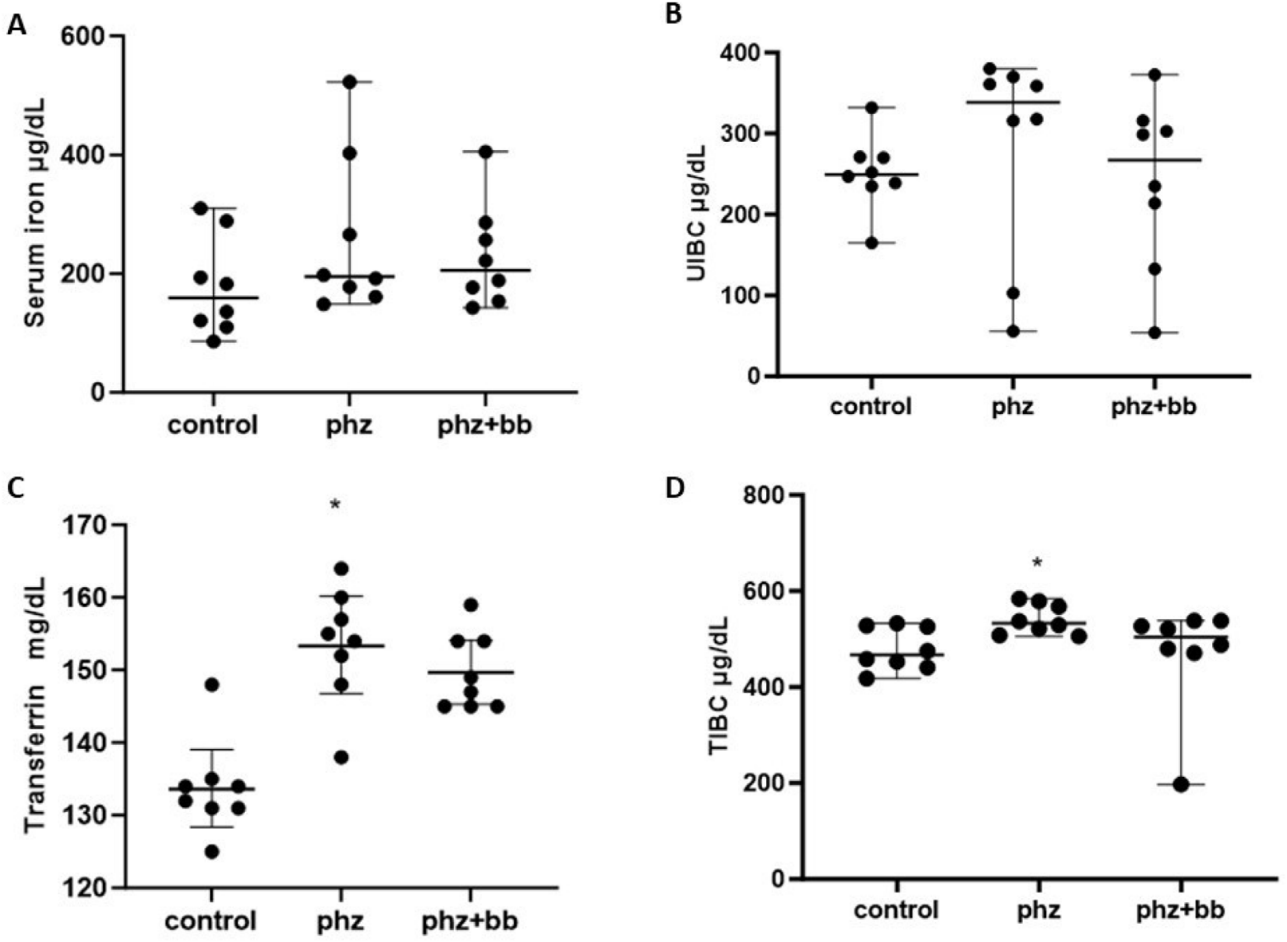
(A) Serum iron (B) Unsaturated iron binding capacity (UIBC) (C) Transferrin (D) total iron-binding capacity (TIBC). Experimental groups: Control, anemia (PHZ), and anemia plus blueberries (PHZ+BB Group). One-way ANOVA complemented by Bonferroni test to compare groups; results expressed as means ± standard deviation. (*) Significant difference compared to the Control Group.

Serum iron and UIBC levels did not differ significantly between the experimental groups (Figure 3A and 3B). Both transferrin and TIBC levels were increased in the PHZ Group when compared to values observed in the Control Group (F-statistic (2, 21) = 7.982; *p*-value = 0.0026). For these parameters, the PHZ+BB Group showed no significant difference from the Control and PHZ Groups (Figure 3C and 3D).

### Acute hemolytic crises cause significant reductions in erythrocytes and increases in total leukocytes

**Figure 4:**
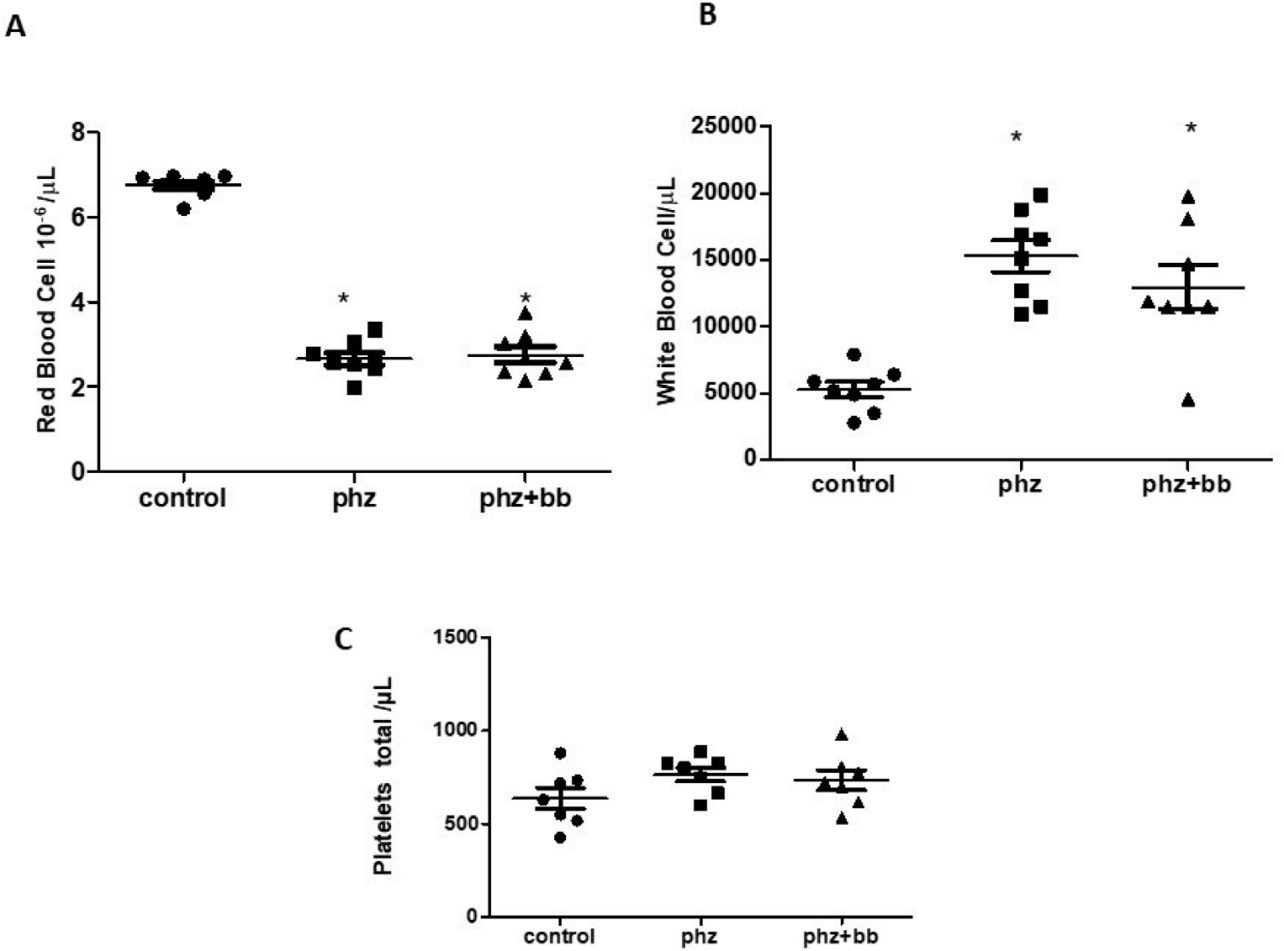
(A) Red blood cell count (B) Total white blood cell count (C) Total platelet count. Experimental groups: Control, anemia (PHZ), and anemia plus blueberries (PHZ+BB). One-way ANOVA complemented by Bonferroni test to compare groups; results expressed as means ± standard deviation. (*) Significant difference compared to the Control Group.

The PHZ and PHZ+BB Groups showed a significant reduction in red cell counts when compared to the Control Group (F-statistic (2, 21) = 246.1; *p*-value < 0.0001 - Figure 4A). Likewise, the PHZ and PHZ+BB Groups showed increases in the white blood cell count compared to the Control Group (F-statistic (2, 21) = 18.42; *p*-value < 0.0001 - Figure 4B). There was no significant difference in the number of platelets between experimental groups (Figure 4C).

### Hemolytic crises induce partial increases in neutrophils in the differential cell count

**Figure 5:**
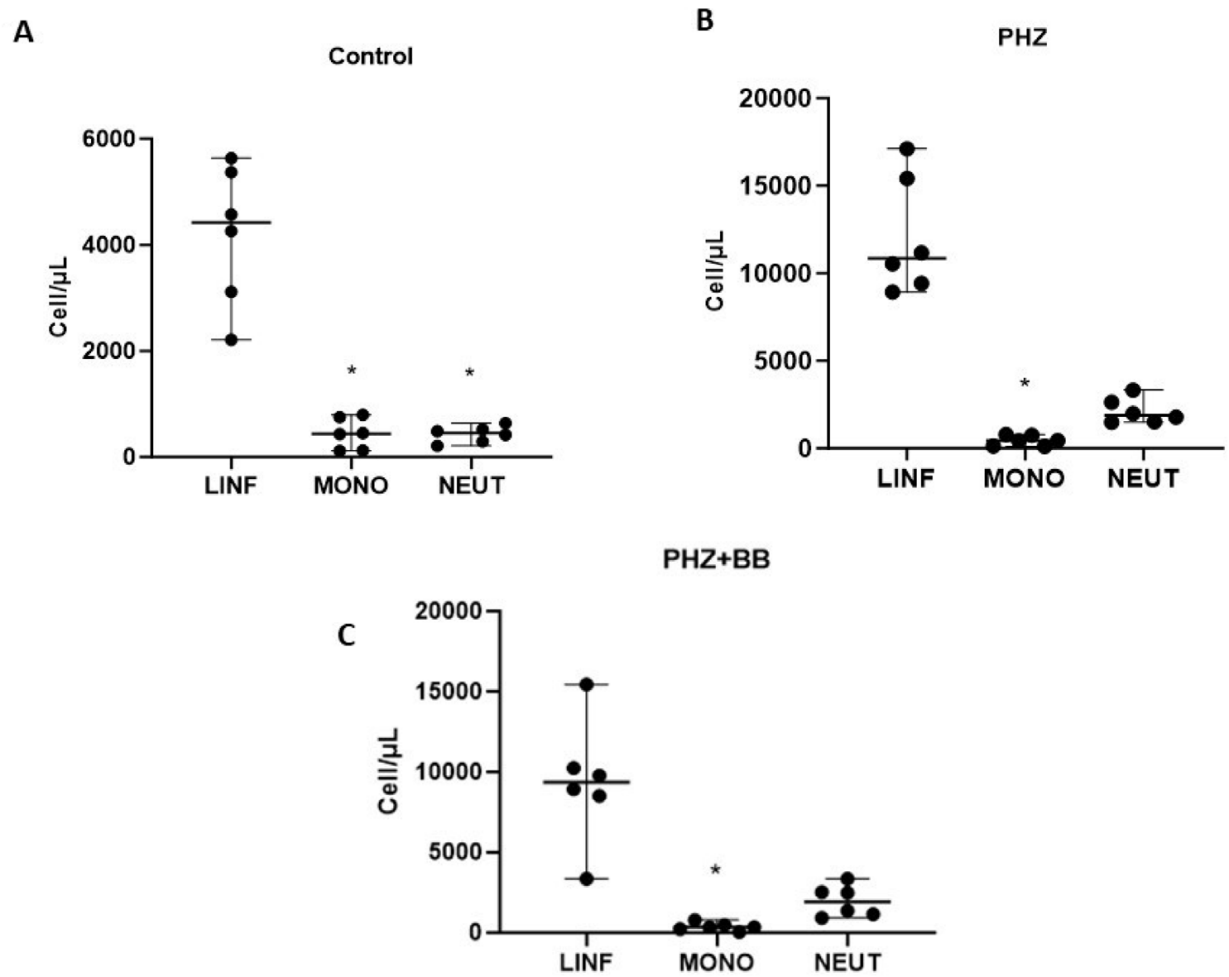
Leukocyte differential percentage count (A) Control Group (B) PHZ Group (C) PHZ+BB Group. Experimental groups: Control, anemia (PHZ), and anemia plus blueberries (PHZ+BB Group). One-way ANOVA complemented by Bonferroni test to compare groups, results expressed as means ± standard deviation. (*) Significant difference compared to the control group.

In the Control Group, the differential leukocyte count showed a significant predominance of lymphocytes in relation to monocytes and neutrophils (F-statistic (2, 15) = 45.81; *p*-value < 0.0001). No significant difference was observed between monocyte and neutrophil cell counts in control animals (Figure 5A). The PHZ and PHZ+BB Groups showed differential leukocyte counts with a significant increase in lymphocytes when compared to monocyte cells alone (F-statistic (2, 15) = 60.00; *p*-value < 0.0001). Moreover, in the qualitative evaluation, the PHZ and PHZ+BB Groups showed reactive lymphocytes. However, in these groups, there was no difference in the number of neutrophils compared to those of lymphocytes and monocytes (Figure 5B and 5C).

### Absolute values of lymphocytes and neutrophils were higher in the group subjected to an anemic crisis

**Figure 6:**
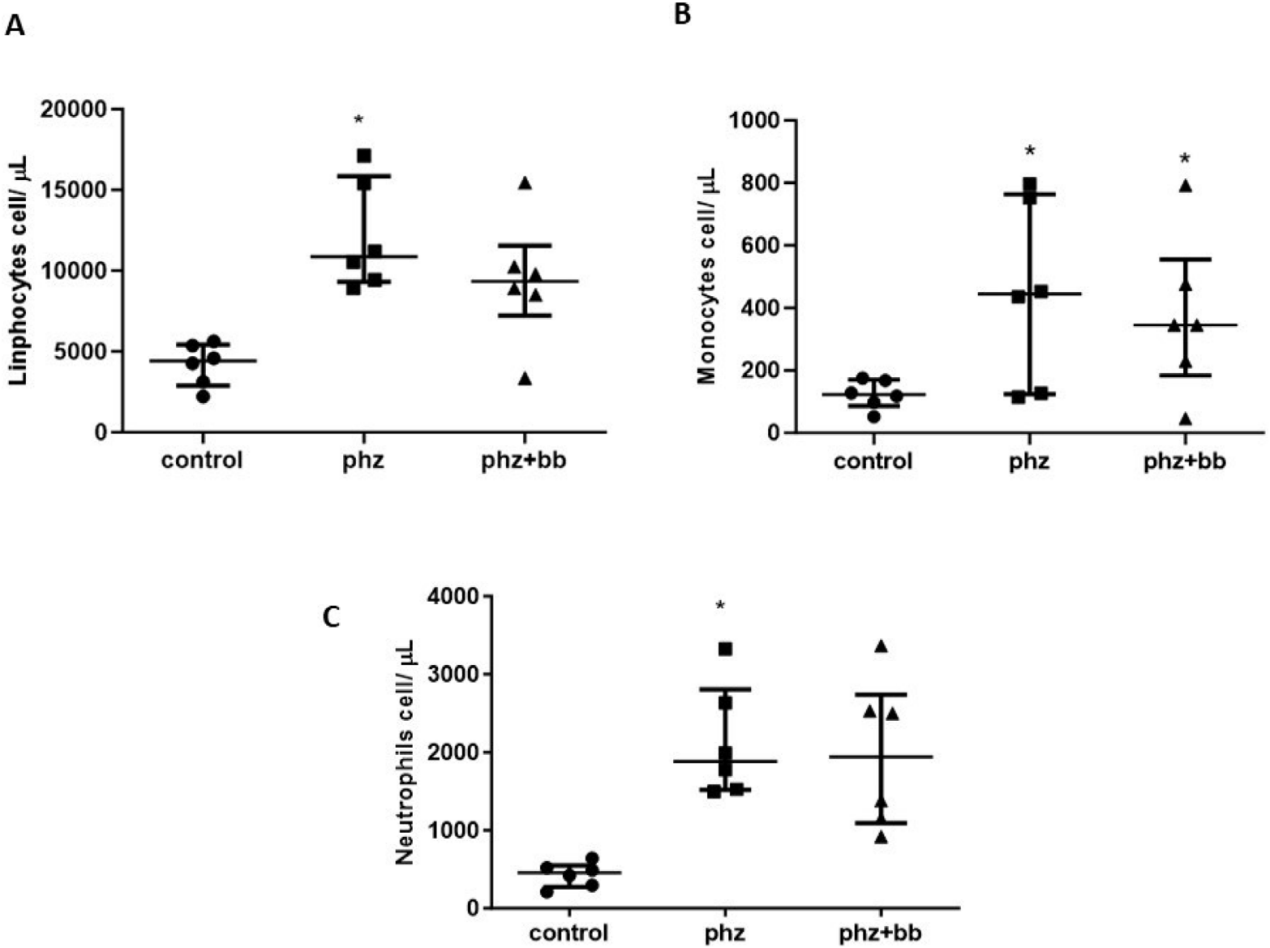
(A) Absolute lymphocyte count (B) Absolute monocyte count (C) Absolute neutrophil count. Experimental groups: Control, anemia (PHZ), and anemia plus blueberries (PHZ+BB Group). One-way ANOVA complemented by Bonferroni test to compare groups; results expressed as means ± standard deviation. (*) Significant difference compared to the control group.

The absolute values of lymphocytes and neutrophils were higher in the PHZ Group than in the Control Group (F-statistic (2, 15) = 10.36; *p*-value = 0.0015); however, in the PHZ+BB Group, the absolute values of lymphocytes and neutrophils showed no differences compared to the Control and PHZ Groups (Figure 6A and 6B). The absolute monocyte count was not different in the PHZ+BB Group when compared with the other experimental groups (Figure 6C).

### IL-10 levels increased in phenylhydrazine-induced anemic crisis

**Figure 7:**
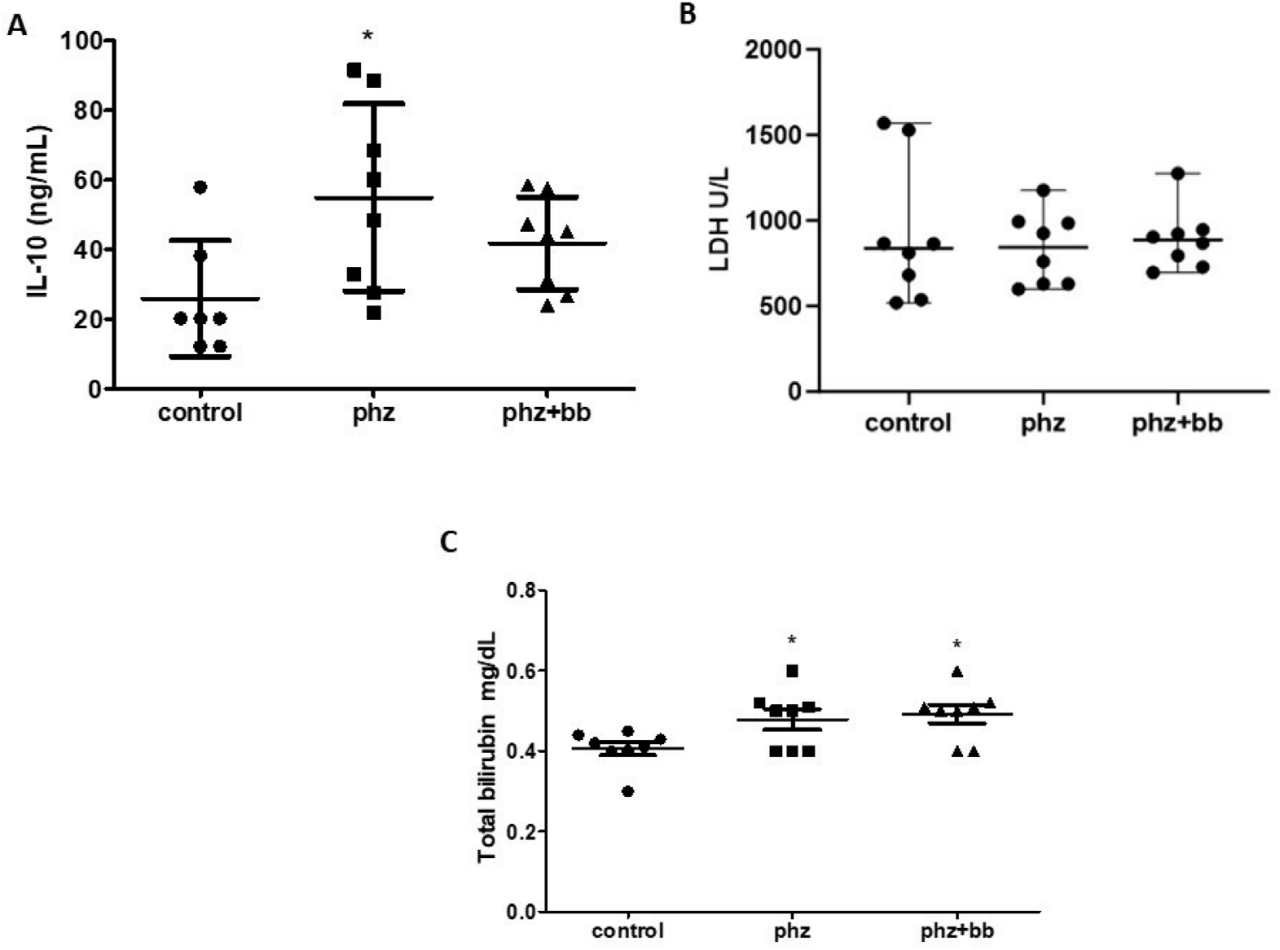
(A) Interleukin −10 (IL-10) (B) Lactate dehydrogenase activity (LDH) (C) Total bilirubin. Experimental groups: control, anemia (PHZ), and anemia plus blueberries (PHZ+BB). One-way ANOVA complemented by Bonferroni test to compare groups; results expressed as means ± standard deviation. (*) Significant difference compared to the control group.

The PHZ Group showed increased IL-10 levels when compared to the control group (F-statistic (2, 20) = 3.971; *p*-value = 0.0353). For this parameter, however, the PHZ+BB Group showed no difference in relation to either the Control or PHZ Groups. Regarding bilirubin levels, the PHZ and PHZ+BB Groups presented increases in total bilirubin concentration compared to the Control Group (*p*-value = 0.0067). The LDH levels did not differ between the three experimental groups (Figure 7A, 7B, and 7C).

### Response of oxidative stress to blueberry treatment

**Figure 8:**
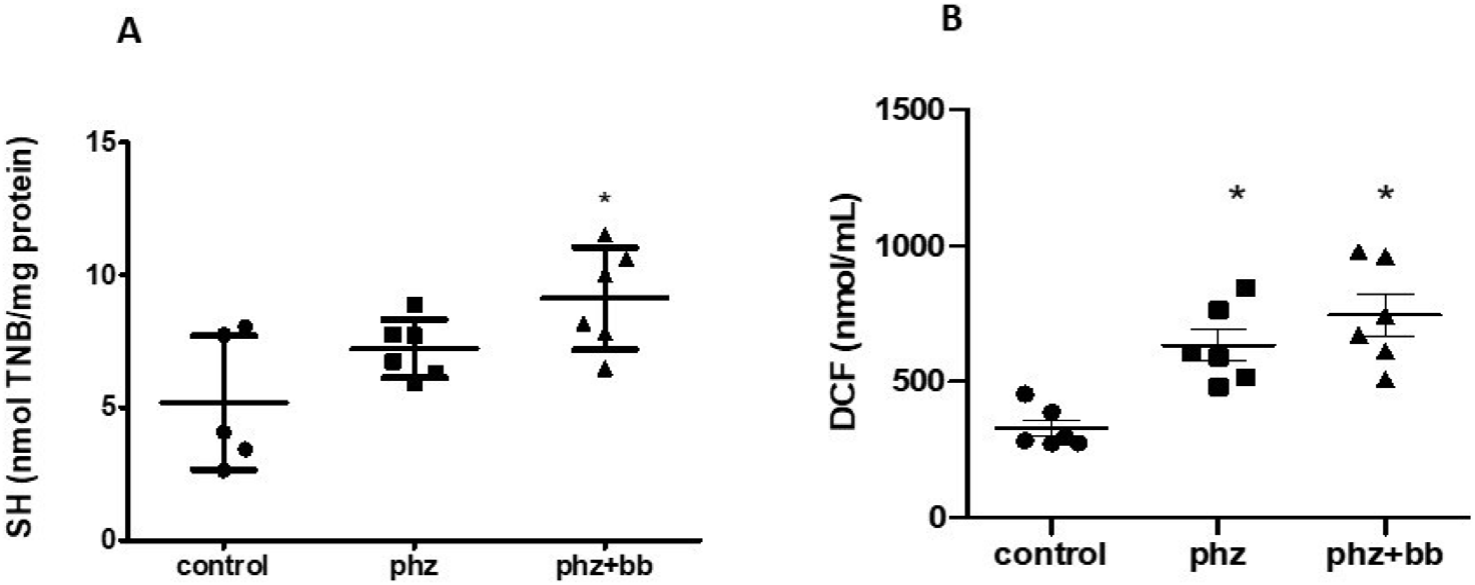
(A) Sulfhydryl group (SH) levels (B) total reactive oxygen species (ROS) (by 2′,7′-dichlorofluorescein levels). Experimental groups: Control, anaemia (PHZ), and anaemia plus blueberries (PHZ+BB). One-way ANOVA complemented by Bonferroni test to compare groups; results expressed as means ± standard deviation. (*) Significant difference compared to the control group.

Blueberry treatment increased total sulfhydryl concentrations in the PHZ+BB group (*p*-value < 0.05 - Figure 8A). However, ROS levels were higher in the PHZ and PHZ+BB Groups when compared to the Control Group (*p*-value < 0.05 - Figure 8B).

## Discussion

**Figure 9:**
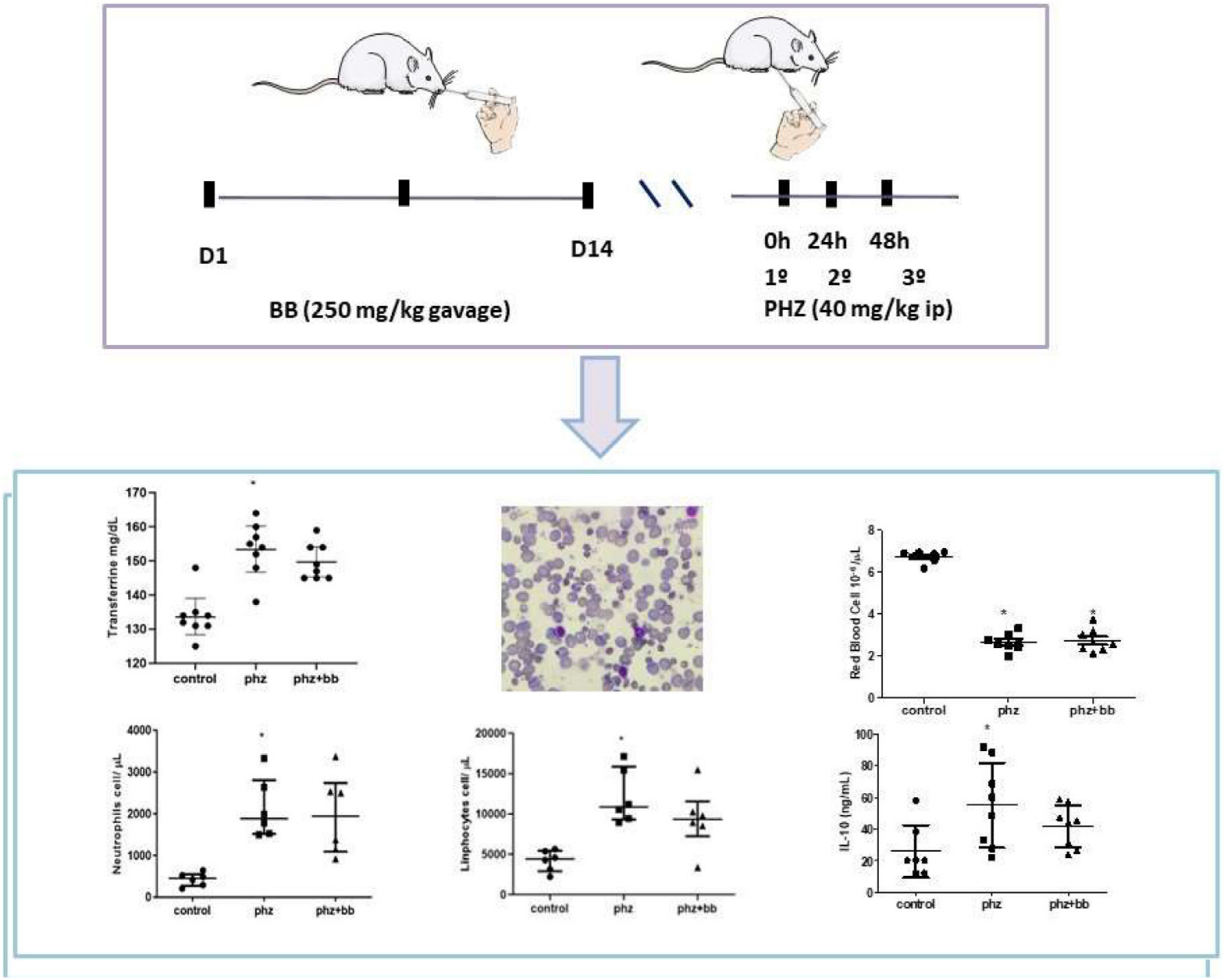
Experimental procedure was based on the establishment of acute hemolytic anemia, through the administration of phenylhydrazine (PHZ), preceded by two weeks of daily treatment with blueberry (BB). This experimental model produces important results regarding the cellular inflammatory response to stress induced by the anemic crisis, such as shown by data on IL-10, neutrophil and lymphocyte counts, as well as relevant changes in erythrocyte morphology and iron metabolism. Parts of the figure were drawn by using pictures from Servier Medical Art. Servier Medical Art by Servier is licensed under a Creative Commons Attribution 3.0 Unported License (https://creativecommons.org/licenses/by/3.0/).”

This is a pioneering study exploring the therapeutic role of blueberries on hematological and inflammatory changes induced by phenylhydrazine. The experimental model of acute hemolytic anemia was successful because both hematological data and spleen morphometric evaluation supported a relevant condition of hemolysis. Furthermore, significant increases in leukocytes and reactive lymphocytes were observed, suggesting an inflammatory response induced by phenylhydrazine. The mechanism underlying this improvement may involve an increase in antioxidant defense represented by the augmented sulfhydryl group levels induced by blueberries. In contrast, blueberry treatment partially modified the differential profile of leukocytes and IL-10 levels. However, the administration of blueberry extract was not effective in mitigating anemia (Figure 9).

A phenylhydrazine-induced acute anemia model was successfully established in this study as the data show a high degree of hemolysis in 0.85% and 0.55% sodium chloride solutions. Furthermore, when analyzing the morphological characteristics of erythrocytes, significant hemolytic characteristics were observed, such as ghost erythrocytes, polychromatophilia, anisocytosis, and platelet aggregation. Phenylhydrazine is known to induce the production of excess hydrogen peroxide, which oxidizes the sulfhydryl groups of proteins, causing their inactivation, peroxidation of membrane lipids, and formation of the product ferrihemochrome, resulting in loss of membrane flexibility, rupture, and consequent intravascular or extravascular hemolysis in the spleen.^18^ Indeed, the data of this study show phenylhydrazine-induced ROS production which could be involved with both oxidative stress and hemolysis. However, blueberry treatment augmented sulfhydryl group levels as a response to oxidative stress. In this sense, blueberry extract was tested because of its known antioxidant and anti-inflammatory properties.^13^ The phenolic compounds from blueberry extract exert a relevant action against oxidative damage in experimental models of pulmonary hypertension and myocardial infarction.^19^^,^^20^ Couto et al.^21^ showed that pterostilbene, a phenolic compound from blueberries, increased cellular viability in H9c2 cells stressed with hydrogen peroxide. Nonetheless, this study shows that erythrocyte loss due to hemolysis was not reduced by blueberry extract administration. This can be demonstrated by the reduction in the number of erythrocytes observed in both groups in which phenylhydrazine was administered, reinforcing the anemic condition of these animals.

Hemolytic crises cause temporary changes in serum iron transport, which can affect the serum levels of the proteins involved in the transport of this metal. Although in this study, alterations in the serum iron levels were not observed in the PHZ Group, an elevated transferrin concentration and total iron-binding capacity were detected in this group. Transferrin is a glycoprotein that preferentially binds iron in its ferric form, preventing oxidation reactions mediated by this metal and promoting its release into cells.^22^ The elevations of TIBC and transferrin implies a counterregulatory response to a hemolytic challenge. On the other hand, these parameters, in the PHZ+BB Group, did not show significant differences when compared to the Control Group or the PHZ Group, suggesting, even if partially, a positive interference of blueberry extract against the harmful consequences of hemolysis and iron release. Similar to this study, Kazmierczak et al.^12^ explored the protective mechanism of polyphenols from blueberry extracts on red blood cells. These authors showed that low molecular weight polyphenols can bind to the polar groups of the erythrocyte membrane and change the chemical properties of the membrane, thereby providing protection against oxidative stress. In this sense, in the PHZ+BB Group, it is possible that there was less iron release due to the protective mechanism of the compounds present in the extract, reducing the need to transport it through the circulation and placing less burden on transferrin function. Further studies are necessary to confirm this hypothesis.

Hemolysis can trigger inflammation because the release of hemoglobin exposes the heme group, which, when free in circulation, can predispose to oxidative and inflammatory processes.^23^ The heme group has been shown to directly participate in the development of diseases associated with red blood cell damage.^24^ It is highly hydrophobic, and according to Kato et al.,^25^ this property is important for activating innate immune cells and inflammatory response. This hypothesis implies that the immune system can detect and respond to hydrophobic substances even if they are not directly associated with pathogens or damage. The concentration of free heme groups arising from hemolysis was not measured directly. In contrast, the total bilirubin level was evaluated as an indirect measure of heme metabolism. According to the data of this study, there was an increase in total bilirubin in both PHZ and PHZ+BB Groups, suggesting augmented levels of circulating heme resulting from hemolysis. Martin-Ventura et al.^26^ found that heme groups amplify neutrophil activity and lead to proinflammatory cellular responses and oxidative stress.

The absolute lymphocyte and neutrophil counts, as well as the total white blood cell count, were increased in the PHZ Group to approximately four times that of the Control Group. These findings indicate activation of leukopoiesis by the bone marrow or the release of these cells from the vascular marginal pool into the circulation. In this context, this increase indicates an inflammatory response to hemolysis.^27^ This process can be mediated by the heme group, which activates protein kinase C and induces the expression of pro-inflammatory cytokines (interleukin-8 and tumor necrosis factor-alpha) and the production of ROS through a process called oxidative burst. In addition, the hemolytic process induces the expression of adhesion molecules and increases the chemotactic activity of neutrophils towards heme gradients.^23^ In contrast, in the PHZ+BB Group, lymphocyte and neutrophil absolute counts showed no significant changes when compared to the Control Group or the PHZ Group. These results suggest that blueberry extract partially attenuates the inflammatory conditions established by hemolysis. Sergazy et al.^28^ showed, in an experimental model of hepatotoxicity induced by CCl4, an anti-inflammatory effect of blueberry extract in the liver by decreasing interleukin-6, tumor necrosis factor-alpha, and interferon-gamma levels. Surprisingly, the data of this study show an increased anti-inflammatory response in the PHZ Group, since IL-10 levels, which are anti-inflammatory cytokines, were augmented in this group compared to control rats. This increase may represent a counter-regulatory response to the inflammatory conditions imposed by the hemolytic crisis experienced in this experimental protocol. However, in the PHZ+BB Group, the levels of this cytokine were not significantly different in relation to the Control Group. This finding may indicate that the components of the extract may have mitigated inflammation in this group. Therefore, there was no evidence of an increase in the cytokine IL-10 in this group, probably because the extract itself reduced the inflammatory response. This finding corroborates with the lower lymphocyte and neutrophil counts in the group treated with the extract.

## Conclusion

In this experimental model, blueberry extract administration decreased neutrophil and lymphocyte counts, and IL-10 levels. This result indicates the mild anti-inflammatory effect of this extract. The data of this study, suggest the antioxidant role of blueberry extract as a mechanism potential. However, the use of blueberries in hemolytic anemia is a non-classical therapeutic approach that requires further study to identify any beneficial effects on blood diseases.

## Data availability

The authors report that the data of this study will be made available without reservation.

## Conflicts of interest

The authors declare no biomedical financial interests or potential conflicts of interest.
